# Comparison of Single Versus Multiple Nonpharmacological Interventions for the Management of Lung Cancer–Related Fatigue: A Systematic Review

**DOI:** 10.1111/crj.70132

**Published:** 2025-10-24

**Authors:** Audrey A. Almassi, Clarice Y. Tang, Sheree Smith

**Affiliations:** ^1^ Faculty of Science, School of Life Sciences University of Technology Sydney (UTS) Ultimo Australia; ^2^ School of Health Sciences Western Sydney University Sydney Australia; ^3^ Institute of Health and Sport Victoria University Melbourne Australia; ^4^ South Western Sydney Local Health District Sydney Australia; ^5^ Faculty of Health and Medical Sciences University of Adelaide Adelaide Australia; ^6^ Greater Brisbane Clinical School, Faculty of Medicine University of Queensland Brisbane Australia

**Keywords:** cancer‐related fatigue, lung cancer, nonpharmacological interventions, quality of life, randomized controlled trials

## Abstract

**Background:**

Lung cancer is one of the common cancers globally. One of the adverse symptoms of lung cancer and its treatment is fatigue. Pharmacological interventions have not shown efficacy on cancer‐related fatigue, and investigations on nonpharmacological interventions may be useful. This systematic review aims to evaluate the efficacy of nonpharmacological interventions on managing fatigue and quality of life outcomes among people undergoing treatment for lung cancer and evaluate if treatment efficacy differed between single and multimodal interventions.

**Methods:**

Relevant literature published in MEDLINE, Scopus, Cochrane Library, CINAHL, and ProQuest from January 2003 to January 2023 was included. Included studies must have: participants over 16 years of age receiving treatment such as chemotherapy, radiotherapy, and/or surgery, cancer‐related fatigue (CRF) as an outcome, and must be randomized controlled trials. Two reviewers independently extracted data from eligible articles, and data analysis was performed using R 4.1.0 software.

**Results:**

Total of 14 randomized controlled trials were included and categorized into four groups: physical activities, traditional Chinese medicine (TCM), education, and dietary counselling. Our extensive search did not find any multimodal studies related to CRF in patients with lung cancer. Pooled results of this systematic review found that TCM and education interventions have a significant positive impact on fatigue in patients with lung cancer. Physical activity and dietary counselling were not effective in managing fatigue. None of the reported nonpharmacological interventions in this review significantly impact QoL.

**Conclusions:**

This review identified that TCM and educational programs improved CRF in patients with lung cancer. However, physical activities and dietary counselling did not show any improvements in fatigue for patients undergoing lung cancer treatment.

**Registration:**

PROSPERO registration ID: CRD42023407326.

## Graphical abstract

- Globally, lung cancer is one of the common cancers and leading causes of death.
- Patients undergo different treatments depending on the type of lung cancer cells.
- Cancer‐related fatigue (CRF) is an adverse symptom in lung cancer patients.
- Cancer related fatigue can be experienced during or after the treatment.
- Nonpharmacological interventions can positively impact reducing and managing CRF.

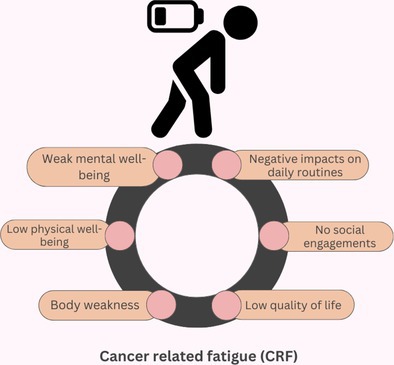

**Abbreviations**
ATP: Adenosine triphosphate
BFI‐C: Brief Fatigue Inventory questionnaire for Chinese
CBT: Cognitive behavioral therapy
CFS‐C: Cancer Fatigue Scale ‐Chinese version
CI: Confidence intervals
CRF: Cancer related fatigue
EORTC QLQ‐C30 Chinese language version: European Organization for Research and Treatment of Cancer Quality of Life Core Questionnaire
FACT‐F: Functional Assessment of Cancer Therapy‐Fatigue
FACT‐L: The Functional Assessment of Cancer Therapies‐Lung
FACT‐L: The Functional Assessment of Cancer Therapies‐Lung
FACT‐LCS: Functional Assessment of Cancer Therapy‐Lung Cancer Subscale
MBSR: Mindfulness‐based stress reduction
MDASI: M.D. Anderson Symptom Inventory
MFSI‐SF: Multidimensional Fatigue Symptom Inventory Short Form
MOE: Margin of error
NCCN: National Cancer Comprehensive Network
NSCLC: Nonsmall cell lung cancer
PE: Psychoeducation
PRISMA: Preferred Reporting Items for Systematic Reviews and Meta‐Analyses
PROSPERO: Prospective Register of Systematic Review
QLQ‐CCC: QoL questionnaire for Chinese cancer patients receiving chemobiotherapy
QoL: Quality of life
RCT: Randomized controlled trial
REM: Random effect model
RoB: Risk of bias
*RPFS*‐*CV*
: Revised Piper Fatigue Scale‐Chinese Version
RVE: Robust variance estimate
SCC: Squamous cell carcinoma
SCLC: Small cell lung cancer
SD: Standard deviation
SMD: Standard mean difference
TCM: Traditional Chinese medicine
TFRS: Tang Fatigue Rating Scale
VAS‐F: Visual Analogue Scale of Global Fatigue, Functional Assessment of Cancer Therapy– Fatigue

## 1 Introduction

Lung cancer is one of the leading causes of death and commonly diagnosed cancers for the last decade [1]. In 2020, 2.21 million new lung cancer cases had been diagnosed, resulting in 1.8 million deaths worldwide [2]. During the past decade, the number of lung cancer cases and mortality rates has decreased because of new technologies associated with screening, early detection, and treatments such as pharmacological and technological‐based therapies or surgery [3]. This means that the survivorship of cancer patients is increasing even though incidence rates of lung cancer continue to be high [3].

While there are different types of lung cancer, the most common types of lung cancer are nonsmall cell lung cancer (NSCLC), small cell lung cancer (SCLC), adenocarcinoma (cancer of glandular cells), and squamous cell carcinoma (SCC) [4]. Depending on the cancer cell type, patients with lung cancer undergo different treatments to reduce the effect of the disease by removing or destroying the cancer cells or tumors [5]. While patients with lung cancer undergo active treatments such as radiotherapy, chemotherapy, or surgery, many patients experience various adverse symptoms [6]. These symptoms can be persistent fatigue, weakness, nausea, hair loss, and weight loss due to appetite changes [6]. Of these symptoms, cancer‐related fatigue (CRF) is the most common symptom reported among patients with cancer [7].

It has been estimated that CRF occurs in 90% of patients during their treatment and between 27% and 80% after treatment [8]. The reported fatigue is unrelated to the specific cancer stage and can occur throughout the entire treatment cycle [9]. National Cancer Comprehensive Network (NCCN) describes CRF as “a distressing, persistent, subjective sense of physical, emotional, and/or cognitive tiredness or exhaustion related to cancer or cancer treatment that is not proportional to recent activity and interferes with functioning” [10]. Ongoing CRF negatively affects patients' quality of life (QOL), social engagements, motivation, and daily activities, which affects their psychological and physical well‐being [11]. Due to the detrimental impact of CRF, it is crucial to introduce appropriate interventions and improve the knowledge of patients with lung cancer about this symptom so they can optimally manage it [12].

Several pharmacological and nonpharmacological interventions have been investigated to manage and reduce the impact of CRF. Pharmacological medications recommended for controlling fatigue symptoms in patients with lung cancer are psycho‐stimulants and hormones such as progesterone, cortisol, dexamethasone, or methylprednisolone, which are ineffective in managing CRF [13]. On the other hand, nonpharmacological interventions have shown improvements in CRF. Several types of nonpharmacological interventions, such as physical activities (yoga, Tai chi, or aerobic exercises), traditional Chinese Medicine (TCM) therapies (acupuncture, acupressure), psychosocial interventions (cognitive behavioral therapy (CBT), mindfulness‐based stress reduction (MBSR), or psychoeducation (PE)), nutritional management, and sleep management, have been recommended [14]. These interventions aimed to alleviate the fatigue symptom through behavioral changes, strengthening muscle tone, and increasing patient energy levels by improving sleep quality, relieving muscular tension, and bringing body and mind into harmony [15]. Such interventions were recommended for reducing and managing fatigue symptoms in patients with lung cancer.

Nonpharmacological interventions such as physical activities have shown positive impacts on fatigue symptoms in patients with lung cancer by improving sleep quality and thereby increasing daytime energy levels to alleviate fatigue symptoms [15]. On the other hand, the efficacy of nonpharmacological interventions can reduce their effect on fatigue among patients with lung cancer who often have comorbidities [16].

Nonpharmacological interventions were suggested to be effective in managing fatigue symptoms in cancer patients, either as a single intervention (single modality) or a combination of interventions (multimodalities). In single‐modal interventions, patients receive only one type of intervention, and in the multimodal model, patients were treated by a combination of nonpharmacological interventions such as acupuncture and yoga to manage the fatigue symptoms during their treatments [14]. Multimodal nonpharmacological interventions have been shown to have a synergistic effect on CRF and help patients manage the adverse symptoms better, increase their energy, and need less amount of time to show their effectiveness as compared to single‐modal interventions [14].

While systematic reviews have been conducted on the effect of interventions on CRF, these reviews often include both patients during treatment and after treatment. However, considering that the effects of nonpharmacological interventions may be reduced during treatment due to the presence of comorbidities and reduction in functional and nutritional status, a systematic review needs to be conducted to evaluate the efficacy of nonpharmacological interventions on CRF among people with lung cancer during treatment.

However, the recent systematic review accomplished by Ma et al. [4] did not specifically focus on the CRF symptoms in patients with lung cancer during treatment or measure their quality of life (QoL). Therefore, given the lack of reviews on this cohort, this systematic review was conducted to study the effect of nonpharmacological interventions on CRF in patients with lung cancer during their treatment and compare the effectiveness of single‐modality and multimodality nonpharmacological interventions on CRF in patients with lung cancer during their cancer treatment.

## 2 Methods

### 2.1 Registration and Guidelines

This systematic review was registered in the International Prospective Register of Systematic Reviews (PROSPERO) registry (CRD42023407326). This review follows the Preferred Reporting Items for Systematic Reviews and Meta‐analyses (PRISMA) 2020 guidance [17] (Table S1). R 4.1.0 software was utilized to analyses systematic reviews and manage the collected data.

### 2.2 Search Strategy

A search was conducted on five databases (MEDLINE, ProQuest, CINAHL, Scopus, and Cochrane Library), including studies published within the last two decades (2003–2023). Studies before 2003 were not included as a clear definition of CRF was not available before that period of time [18].

The key search terms were identified using the PICO framework [19]—“lung cancer”, “fatigue”, “physical exercise”, “nutrition”, “complementary therapi*”, “acupuncture”, and “non‐pharmacological intervention*” (Table S2–S6). These terms were truncated, and Boolean operators were used to ensure capturing of all necessary data. In addition, the first author (AA) performed manual reference list screening for additional studies from identified published studies.

### 2.3 Study Selection

The inclusion criteria for this systematic review are as follows: 1) all studies included participants who were receiving treatment for lung cancer, which included chemotherapy, radiotherapy, and/or surgical treatments, 2) participants must be over 16 years of age, 3) CRF was the outcome measured in the studies, and 4) all studies must be randomized controlled trials. Studies that included participants who had completed their lung cancer treatment were excluded from the review. In addition, the grey literature with incomplete results, study protocols, and non‐English publications were excluded.

After removing the duplicates using Covidence software, two researchers (AA and CT) independently screened the titles and abstracts of the full‐text studies.

Any conflicts and disagreements were resolved by the third researcher (SS). For the next phase of this review, two researchers (AA and SS) reviewed the full‐text articles to assess their eligibility. Any conflicts and disagreements were resolved by the third researcher (CT).

### 2.4 Data Extraction

Two researchers (AA and CT) extracted the data from the eligible articles using a review‐specific data extraction sheet (Table S7). The following data were extracted: authors, publication year, country, number of participants, age, age mean in control and intervention groups, gender, type of lung cancer, type of treatment, measurement tools for fatigue and QoL, type of intervention, single or multimodality, dosage, duration, trained staff, blinded or nonblinded study, completion or withdrawal rates, and outcome measurements for control and intervention groups.

### 2.5 Methodological Quality Evaluation

Researchers (AA and SS) independently evaluate the methodological quality of the eligible studies by using Version 2 of the Cochrane risk‐of‐bias tool for randomized trials (*RoB 2*) [20]. This assessment tool evaluates the risk of bias in various study domains such as sequence generation, allocation concealment, blinding of participants and personnel, blinding of outcome assessors, incomplete outcome data, selective outcome reporting, other biases, and overall risk of bias. Studies were judged in each domain as having a low, unclear, or high risk of bias. The overall risk of bias was judged as moderate, serious, or critical risk of bias and shown in Table 2.

### 2.6 Data Analysis

In systematic review research, complex data structures report the effectiveness of the interventions [21]. For this purpose, this systematic review utilized R 4.1.0 software to analyses the data. The forest plots were created to represent the effect sizes, SMD (standard mean difference) was calculated as fatigue, and QoL measurement tools utilized different scales [22]. For calculating the effect sizes in this review, SMDs for fatigue, QoL and follow‐ups with 95% CI (confidence intervals) were applied. For retrieving SDs in studies that did not provide SD values, the first margin of error (*MOE*) value was calculated (upper CI–lower CI) and then the sample size (*N*), mean, and *MOE* values were inserted into the formula $\frac{\mathrm{MOE}\times \surd N}{1.96}$ [23].

The SMD values are categorized into the following three groups: 0.2–0.5, considered as small size; 0.5–0.8, considered medium size; and > 0.8, considered large size. The results will be considered statistically significant if the *p* < 0.05 [24]. For evaluating the heterogeneity between the collected studies, the funnel plot and random effect model (REM) were conducted to measure the *I* ^2^, tau^2^, tau, and *H* ^2^.

*I* ^2^ values can be categorized into moderate heterogeneity (30%–60%), substantial heterogeneity (50%–90%), and considerable heterogeneity (75%–100%) [25].

Egger's regression test was carried out to obtain the publication bias [26].

## 3 Results

**FIGURE 1:**
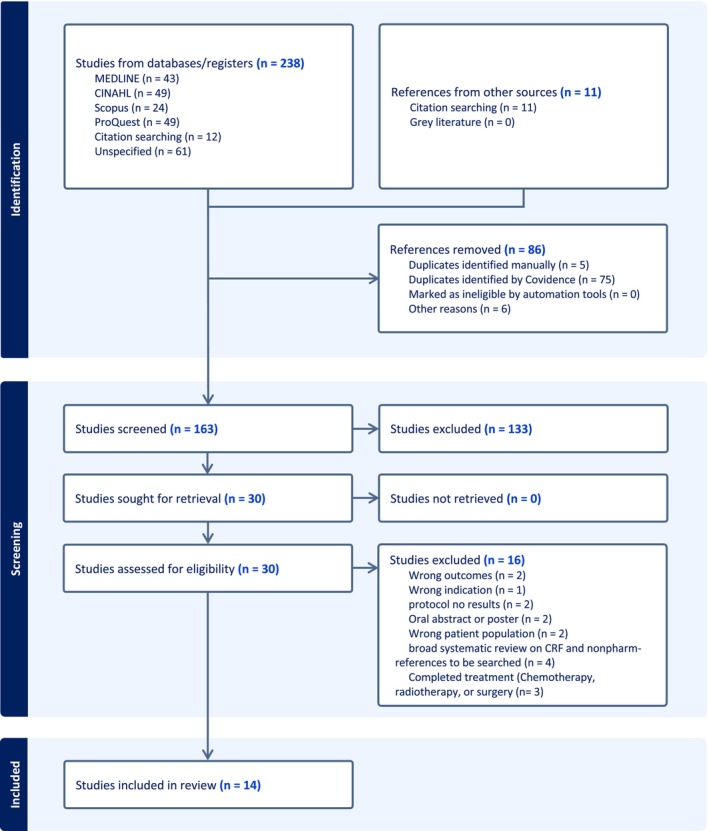
PRISMA flow diagram.

The PRISMA flow diagram is presented in Figure 1 representing the literature search retrieval process and the number of studies identified at each stage of the review process. From the selected databases, 238 studies were collected, and 11 studies were from the references of other systematic reviews. After screening the title and abstract for eligibility, 30 studies remained, of which 16 studies were excluded because of different reasons such as wrong patient population, a protocol that did not have results, wrong outcomes, and oral abstract or poster. Fourteen studies were eligible to be included in this systematic review.

### 3.1 Study Characteristics

The included studies were published between 2010 and 2022 and reported in Table 1. A total of 982 participants were included across the 14 studies, with sample sizes ranging between 15 and 140 participants per study. Four studies were pilot randomized control trials [27, 28, 29, 30], and 10 were randomized control trials [31, 32, 33, 34, 35, 36, 37, 38, 39, 40]. Ten studies were conducted in Asian countries such as China, Taiwan, Korea, and Thailand [27, 28, 31, 32, 33, 34, 35, 36, 37, 38], two in Denmark and the USA [29, 39], and two studies were conducted in Australia [30, 40]. Participants were mixed in both sexes (male and female), and the proportion of males to females was 1.5:1. For the cancer type, two studies did not determine the type of lung cancer, and one study only mentioned advanced lung cancer. Six studies included participants with NSCLC while five included participants with either NSCLC or SCLC. All 14 studies only included single‐modal interventions, ranging between 2 and 20 weeks with a follow‐up period between 2 weeks and 6 months.

**TABLE 1:** Study characteristic table.

Author/publication year/country	Study design	No. participants (M/f)	Age (control group mean/intervention group mean)	Cancer type	Treatment type	Type of intervention	Duration (weeks)	Fatigue assessment tool	Quality of life assessment tool
Cheng, 2017, China	RCT	28 (13/15)	62/58	NSCLC	Chemotherapy or radiotherapy	Acupuncture	4	BFI‐C	FACT‐LCS
Lin, 2019, China	RCT	100 (59/41)	61.85/59.5	NSCLC	Chemotherapy	Auricular acupressure	9	CFS‐C	—
Jeong, 2010, Korea	Pilot RCT	40 (15/25)	53.4/49.4	N/A	Chemotherapy or radiotherapy	Bojungikki‐tang	2	VAS‐F	FACT‐F
Kiss, 2016, Australia	Pilot RCT	24 (12/12)	64.3/62.6	NSCLC or SCLC	Chemotherapy and/or radiotherapy	Intensive nutrition program	12–13	FACT‐L	FACT‐L
Tang, 2014, Taiwan	Pilot experimental	57 (33/24)	66.1/62.6	NSCLC or SCLC	Chemotherapy	Acupressure	20	TFRS	—
Tan, 2019, China	RCT	94 (65/29)	N/A	NSCLC or SCLC	Chemotherapy	Cognitive education	12	MDASI	—
Hwang, 2012, Taiwan	RCT	24 (12/12)	58.5/61	NSCLC	Targeted therapy	Exercise training	8	EORTC QLQ‐C30	EORTC QLQ‐C30
Wangum, 2013, Thailand	RCT	60 (41/19)	57.3/54.8	N/A	Chemotherapy	Multidisciplinary education	9	Piper Fatigue Scale	—
Chan, 2011, China	RCT	140 (116/24)	N/A	Advanced lung cancer	Radiotherapy	Psychoeducation	6	Piper Fatigue Scale	—
Egegaarda, 2019, Denmark	RCT	15 (5/10)	65/64	NSCLC	Radiotherapy	Daily exercise training	7	Study specific scale	FACT‐L
Xu, 2022, China	RCT	126 (57/69)	56.2/56.6	NSCLC	Chemotherapy	Moxibustion	3	RPFS‐CV	QLQ‐CCC
Zhang, 2016, China	RCT	91 (68/23)	−/62.8	NSCLC or SCLC	Chemotherapy	Tai‐chi	12	MFSI‐SF	—
Dhillon, 2017, Australia	RCT	111 (60/51)	64/64	NSCLS or SCLC	Chemotherapy and targeted therapy	Physical activity	8	FACT‐F	EORTC QLQ‐C30
Hoffman, 2017, USA	Pilot RCT	72 (32/40)	65.6/67.4	NSCLC	Surgery	Physical activity	6	BFI	—

**TABLE 2:** Risk of bias assessment.

Author, year	Sequence generation	Allocation concealment	Blinding of participants and personnel	Blinding of outcome assessors	Incomplete outcome data	Selective outcome reporting	Other sources of bias	Overall risk of bias
Cheng, 2017	Low	Unclear	Low	Low	Low	Unclear	High	Low
Lin, 2019	Low	Low	Unclear	Unclear	Low	Unclear	Unclear	Moderate
Jeong, 2010	Low	High	High	High	Low	Unclear	Unclear	High
Kiss, 2016	Low	Unclear	High	Unclear	Unclear	Low	High	High
Tang, 2014	High	Unclear	High	Low	Unclear	Unclear	Unclear	Moderate
Tan, 2019	Low	Low	Low	Low	Unclear	Unclear	Unclear	Low
Hwang, 2012	Low	Low	High	Low	Unclear	Low	High	Moderate
Wangum, 2013	Low	Unclear	Unclear	Unclear	Unclear	Unclear	Unclear	Moderate
Chan, 2011	Unclear	Unclear	Unclear	Low	Low	Unclear	High	Moderate
Egegaarda, 2019	Low	Low	Unclear	Unclear	Unclear	Unclear	High	Moderate
Xu, 2022	Low	Unclear	High	High	Unclear	Unclear	Unclear	High
Zhang, 2016	Low	Low	High	High	Unclear	Unclear	Low	Moderate
Dhillon, 2017	Low	Low	Low	High	Low	Unclear	Unclear	Moderate

### 3.2 Fatigue Measurements

All studies measured fatigue using validated scales except for one study [39] which asked participants to self‐report their fatigue in free text [39]. The most frequent assessment tools were fatigue instruments: BFI and Piper Fatigue Scale [29, 31, 33, 35, 36]. A total number of four studies reported the follow‐ups after intervention completion. Three studies reported follow‐up of study participants [30, 33, 34, 40] with a single follow‐up across a range of time frames between 2 weeks and 6 months. Only one study [40] had two follow‐up time points (4 and 6 months).

### 3.3 Quality of Life Evaluation

Apart from fatigue measurements, seven studies [28, 30, 31, 33, 35, 39, 40] also evaluated the participants' QoL before and after intervention.

### 3.4 Adverse Events

Two studies [33, 40] reported minor adverse events such as musculoskeletal pain, vomiting, fever, bleeding, anorexia, and nausea.

### 3.5 Control Group

All studies had control groups that received usual care, which consisted of unsupervised exercises, low‐impact exercises, education, and use of placebo (sham needles).

### 3.6 Intervention Categories

All identified studies used single‐modal interventions to treat CRF. The interventions used were clustered into four groups: physical activity (*n* = 5), TCM (*n* = 5), nutritional interventions (*n* = 1), and education (*n* = 3).

### 3.7 Quality Assessments of Studies

Quality of 14 studies was assessed for risk of bias by using the Rob 2 tool. Overall, the quality of studies was moderate. All studies provided sufficient information related to sequence generation, blinding participants and personnel, and blinding of outcome assessors. However, six studies did not report sufficient information on allocation concealment. Eight studies provided unclear, incomplete outcome data. Twelve studies reported insufficient information in the selective outcome reporting section, and seven had a lack of information in other sources of bias.

Three studies provided the necessary information for blinding participants and personnel methods; four studies did not mention their blinding methods [28, 34, 36, 39]. For the blinding outcome assessors' section, six studies reported sufficient information.

### 3.8 Physical Activity Interventions

**FIGURE 2:**
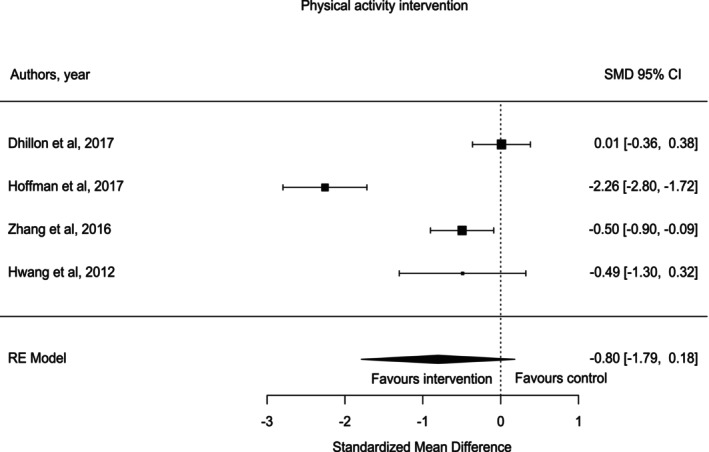
Physical activity intervention forest plot.

Physical activity was a commonly used nonpharmacological intervention for managing adverse symptoms such as fatigue in patients with lung cancer. Physical activities were categorized into different types, such as supervised exercises, tai chi, aerobic exercises, and walking. In this review, four studies [29, 31, 32, 40] applied different types of physical activities to observe the effect of them on managing CRF in patients with lung cancer while they underwent treatments. Duration of interventions varied: 6 weeks [29], 7 weeks [39], 8 weeks [31, 40], and 12 weeks [32]. One study [39] was excluded from the meta‐analysis of physical activity interventions' outcomes due to participants self‐reporting and the unavailability of exact reported data. Overall, the effect of physical activity on CRF while people were undergoing cancer treatment was not significant (SMD = −0.8, 95% CI [−1.79 to 0.18]) (Figure 2).

### 3.9 Traditional Chinese Medicine Interventions

**FIGURE 3:**
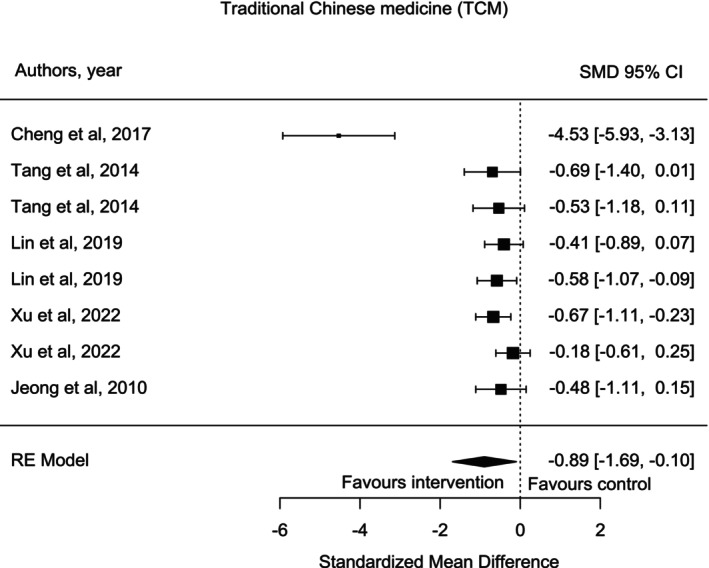
Traditional Chinese Medicine intervention forest plot.

Studies associated with patients with lung cancer utilized traditional Chinese medicine (TCM) interventions such as acupuncture, acupressure, or moxibustion to manage their fatigue symptoms. Five studies utilized TCM to evaluate their effects on patients with lung cancer symptoms [27, 28, 33, 35, 37]. Three studies [27, 35, 37] had two intervention groups and one control group. Interventions were conducted over differing time frames such as 2 weeks [28], 3 weeks [35], 4 weeks [33], 9 weeks [28], and 20 weeks [27]. As presented in Figure 3, studies cumulatively favored intervention on CRF in participants in comparison to the control group (SMD = −0.89, 95% CI [−1.69, −0.10], *I* ^2^ = 93.67%).

### 3.10 Educational Interventions

**FIGURE 4:**
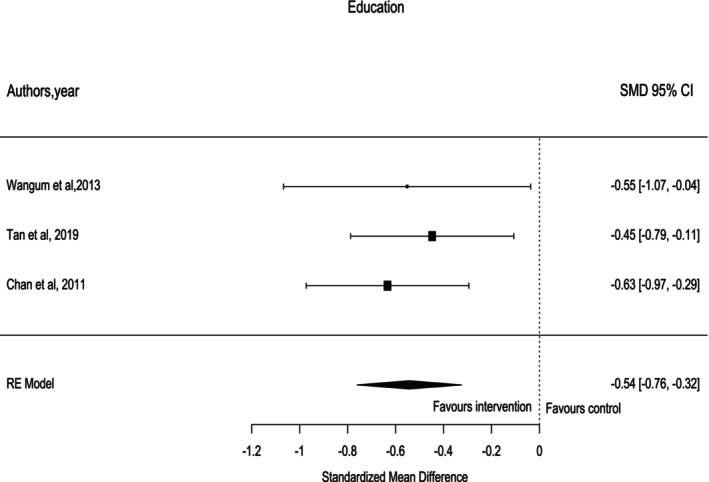
Education intervention forest plot.

Three studies [34, 36, 38] reported the efficacy of different educational programs in managing fatigue in patients with lung cancer. These educational programs included psychoeducation delivered by a single health professional [34], multidisciplinary education [36], and cognitive education [38]. The duration of each educational intervention varied from 6 to 9 to 12 weeks, respectively. As shown in Figure 4, the cumulative result of the three studies indicated that education interventions improve CRF when compared to the control group (SMD = −0.54, 95% CI [−0.76, −0.32], *I* ^2^ = 0%).

### 3.11 Nutritional Intervention

In this review, only one study (*N* = 24) [30] met the inclusion criteria and represented the effectiveness of dietary counselling for managing CRF. This study utilized the intensive nutrition program for the participants and consisted of individual dietary counselling and dietitians controlling the enteral feeding and giving nutritional advice for managing symptoms. The result of the study [30] represented that it favors control as the sample size was small and there was no difference between the control and intervention groups.

### 3.12 QoL Assessments

The QoL of the participants was evaluated in seven studies [28, 30, 31, 33, 35, 39, 40] and divided into the following three intervention clusters: physical activities, TCM, and dietary counselling, respectively.

#### 3.12.1 QoL and Physical Activity

As shown in Figure S1, three studies assessed the QoL of the participants after completing the intervention. Two studies [31, 39] had a small sample size (*N* = 24 and *N* = 15) and large CI, representing low precision. The cumulative result showed that physical activity did not result in a statistically significant improvement in QoL when compared to the control group (SMD = 0.15, 95% CI [−0.38, 0.68]).

#### 3.12.2 QoL and Traditional Chinese Medicine

Figure S2 represents three studies [28, 33, 35] that evaluated the QoL in participants who received TCM interventions. One pilot randomized control trial study [28] was included in the evaluation, which had a small sample size (*N* = 40) and large CI. The total result for the evaluation of TCM on the QoL of the participants in three studies showed that TCM was not effective on the QoL of the participants when compared to controls (SMD = 0.91, 95% CI [−0.25, 2.07]).

#### 3.12.3 QoL and Nutritional Interventions

One study [30] reported the QoL in the participants after completing the intensive nutrition program. The study was a pilot RCT with a small sample size (*N* = 24) and large CI, indicating low precision in the result. The results from this study found that dietary counselling was not effective in improving QoL of participants when compared to control groups (MD = 6.7, 95% CI [−0.47, 1.15]).

### 3.13 Postintervention Follow‐Up for QoL

Four studies [30, 33, 34, 40] reported postassessment follow‐ups (Figure S3). The duration of follow‐ups was diverse, between 2 weeks and 6 months. One study [40] had two follow‐up assessments (4 and 6 months). Two studies [30, 33] had small sample sizes (*N* = 28, *N* = 24) and large CI ranges. All studies demonstrated consistent results that the interventions were ineffective during the follow‐up times. Although the results favored intervention, they were not statistically significant (SMD = −0.09, 95% CI [−0.03, 0.14]).

### 3.14 Publication Bias

To ensure an assessment of publication bias, a funnel plot and Egger's test were completed (Figure S4). The funnel plot and Egger's test demonstrated that publication bias (*p* = 0.5468) was not evident.

## 4 Discussion

This systematic review identified four different clusters of nonpharmacological interventions that have a role in reducing and managing CRF in patients with lung cancer and found that both TCM and education were effective in treating CRF among people receiving treatment during lung cancer. Physical activity and nutritional interventions were found to be ineffective.

Nonpharmacological interventions can be divided into four separate clusters. The first cluster is physical activities such as tai chi or exercise activities. Physical activities have been introduced as first‐line interventions for managing CRF in patients with lung cancer since the last decade [41]. Zhang et al. [32] demonstrated tai chi for 12 weeks and achieved positive results in decreasing the CRF symptoms and increasing vigor in patients with lung cancer during their chemotherapy.

However, our pooled physical activity results showed no effect on CRF during cancer treatment. The lack of positive results favoring physical activity may be due to a multitude of factors [31]. Factors such as the intensity and type of physical activities play a crucial role and can influence the impact of CRF on patients with lung cancer during their treatment [16]. Furthermore, concomitant diseases such as COPD in patients with lung cancer can have a negative impact on physical activities and worsen their fatigue symptoms [16]. This review's findings clearly show that the varied duration and type of physical activities meant that exercise programs were of different intensity and duration, which could have impacted their efficacy, especially since a reduction in fatigue has been found most often in studies that use lower intensity exercises for longer durations. Two studies (Hoffman and Hwang) had smaller sample sizes and hence larger CIs, so they were considered less accurate than the other two studies [31, 41]. In addition, the lack of reporting information from one study [39] could have impacts on the overall result of this intervention.

The cluster associated with TCM interventions reported the efficacy of these interventions in managing lung cancer‐related fatigue. The pooled results showed that TCM significantly impacts fatigue in patients with lung cancer. In TCM, it is believed that adverse symptoms are caused because of *Qi* (life energy) and *Yin* and *Yang* imbalance [42]. Hence, it is crucial to regulate the viscera and tonify *Qi* and blood to improve CRF in patients [42]. Acupuncture, acupressure, and moxibustion help to restore the natural flow of *Qi*, rebalance the *Yin* and *Yang*, and relieve the discomfort from the body, which is related to body energy [33]. Bojungikki‐Tang also positively impacts fatigue as it tonifies *Qi* and follows the TCM theory [28]. Bojungikki‐Tang assisted with the activation of the immune system by increasing the lymphocyte cell surface antigens and treated CRF by inhibiting TNF‐α, IL‐10, INF‐γ, IL‐6, and TGF‐β1 in patients [27]. As the results show, TCM methods represent promising results for lung cancer fatigue and can be efficient methods for mitigating adverse symptoms.

Furthermore, Molassiotis et al. [43] reported that cancer‐related fatigue in patients improved by using acupuncture up to 36% after completing chemotherapy sessions. The most common acupoint is ST36, which is widely used in acupuncture, acupressure, and moxibustion studies [44]. ST36 helps patients manage their fatigue symptoms as it upregulates the level of ATP synthesis in the body and increases the integration level of mitochondrial ATP when the body feels tired [44]. Acupressure is another TCM method that improves the CRF by 19% and does not need to use needles or trained personnel, which makes it pleasant and feasible for patients with reduced potential for adverse events [43]. One possible reason that TCM studies showed overall positive results can be related to large sample sizes and more accurate reporting information by the experts and participants.

In this review, educational interventions have been found to be effective on CRF among patients with lung cancer. This finding is supported by the current literature where educational interventions such as cognitive behavioral therapy have shown improvements in cancer‐related fatigue [44]. Du and colleagues found that educational program interventions improved fatigue symptoms in patients as they gained sufficient knowledge about the coping and managing strategies for these adverse symptoms [45]. Furthermore, patients are more likely to participate in educational programs as these programs assist them in managing fatigue symptoms. These interventions' delivery mode and duration significantly impact their efficacy in managing adverse symptoms in patients with lung cancer [4]. In agreement with our review, Poort et al. [46] showed that 12 weeks of CBT intervention affects CRF and improves cancer patients in the short term. In addition, Mustain et al. [47] found that psychoeducational and CBT interventions have significant effects on CRF in patients who are receiving treatments, and the effect can continue for a few months after the completion of the intervention.

Malnutrition is caused by cancer treatments and adverse symptoms such as loss of taste or appetite [48]. Cancer‐related fatigue in patients with cancer may be improved by nutritional supplements high in fruits, whole‐grain products, vegetables, and anti‐inflammatory fatty acids [48]. Personal dietary programs did not influence the intervention group in dietary counselling intervention, as Kiss et al. [30] study reported. Although it did not show positive effects, a randomized control trial with a larger sample size would be needed to confirm the pilot result and observe the effectiveness of dietary counselling on cancer‐related fatigue.

Quality of life has been shown to reduce during cancer treatment as patients may experience treatment difficulties such as dyspnea or fatigue, which often makes them feel more isolated and less motivated to engage in any activities and experience more emotional difficulties [11]. Unlike previous reviews [49, 50] that have found that nonpharmacological interventions were effective in improving QoL, the findings from this review did not indicate that interventions improved QoL among patients with lung cancer. It may be plausible that the lack of positive findings could be due to the lack of long‐term follow‐up amongst studies in this review. Quality of life measures require longer time periods and/or prolonged intervention rather than treatment durations to result in a positive change [28].

Some studies investigate the effects of nonpharmacological interventions in posttreatment follow‐ups [30, 33, 34, 40]. Overall, posttreatment follow‐ups did not achieve favorable outcomes as the duration of follow‐ups differed in each study and therefore could not establish an effect. Duration in posttreatment follow‐up plays a vital role for postassessments as the effect of the intervention can decrease due to patients' loss or withdrawal because of personal reasons or death, changes in treatment regimen, or loss of motivation in patients to control the effects of the intervention during follow‐up [40].

Patients with lung cancer can consider TCM as a practical and feasible intervention for managing fatigue symptoms during their treatments. The included studies reported minor adverse events, which show the safety of these interventions. Educational interventions also showed positive results as they can improve patients' knowledge of self‐care and managing strategies for disease symptoms. Dietary counselling and physical activities were shown to be ineffective in managing fatigue in patients with lung cancer during their treatments. However, the efficacy of nonpharmacological interventions on CRF appeared to be short‐lived because of a lack of posttreatment follow‐ups.

There are some limitations in this systematic review. First, there was no multimodality nonpharmacological intervention study to investigate the effect of it on cancer‐related fatigue in patients with lung cancer. Therefore, this review only included single‐modality nonpharmacological interventions. It is crucial to evaluate multimodal nonpharmacological interventions that can reduce CRF in patients with lung cancer as combining a variety of modalities may be of benefit to patients at different times across their treatment trajectory.

Secondly, included studies' participants had high variability of characteristics such as different assessment tools, types of intervention, durations, and the stage and type of lung cancer.

This can have effects on the results of the meta‐analysis and favors the results for the control and shows nonpharmacological interventions on CRF to be ineffective.

Thirdly, the exclusion of the non‐English published studies is another limitation, which means some studies have been omitted and not included in this systematic review. This can have impacts on meta‐analyses results.

For future researchers, it is crucial to perform more research with larger trial groups and longer period of time to achieve better results about the effectiveness of different types of nonpharmacological interventions on CRF in patients with lung cancer and observe the impact of the multimodal interventions on managing this symptom in patients with lung cancer. Adaptive trials may provide a future opportunity to investigate the effectiveness of the single‐modal versus multimodal interventions as a more person‐centered approach in the provision of nonpharmacological interventions for patients with lung cancer group and condition.

## 5 Conclusions

This systematic review provided more information about commonly used nonpharmacological interventions, their impact on CRF and on quality of life of patients with lung cancer who undergo treatments. The included studies in this review applied single‐modality nonpharmacological interventions for managing fatigue in patients with lung cancer during treatment.

As the results of this systematic review represented, TCM and educational programs showed positive effects on cancer‐related fatigue, while physical activities and dietary counselling programs did not show a significant impact on CRF in patients with lung cancer. None of the included nonpharmacological interventions in this review significantly impact the quality of life of patients; therefore, further research is needed to achieve accurate results.

In conclusion, it is essential to validate the findings of this review through studies with larger sample sizes, as the relatively small sample sizes in the included studies limit the generalizability and reliability of the results. The outcomes of the individual analyses, therefore, provide only preliminary insights into the effectiveness of nonpharmacological interventions, highlighting the need for further research to confirm these findings.

## Author Contributions

Audrey A. Almassi, Australia (audrey.almassi@student.uts.edu.au) is involved in performing the research, collecting data, analyzing data, writing the draft, reviewing and editing the manuscript. Clarice Y. Tang, Australia (clarice.tang@vu.edu.au) is involved in conceptualization, data analysis, review, and editing the manuscript and supervision. Sheree Smith, Australia (sheree.smith@adelaide.edu.au) is involved in conceptualization, data analysis, review, and editing the manuscript and supervision. Audrey Almassi (AA), Prof. Clarice Tang (CT), and Prof. Sheree Smith (SS) contributed to the conception, acquisition, analysis, and drafting of the manuscript. We approved the final version of this manuscript.

## Ethics Statement

Ethical approval was not required for this review as it did not involve human participants, animal experiments, or identifiable personal data.

## Conflicts of Interest

The authors declare no conflicts of interest.

## Supporting information


**Table S1:** PRISMA 2020 checklist.
**Table S2:** Cochrane library search table.
**Table S3:** MEDLINE search table.
**Table S4:** Scopus search table.
**Table S5:** CINHAL search table.
**Table S6:** ProQuest search table.
**Table S7:** Data extraction sheet template.
**Figure S1:** QoL assessment after completion physical activity interventions.
**Figure S2:** QoL assessment after completion traditional Chinese medicine (TCM) interventions.
**Figure S3:** Intervention post assessment.
**Figure S4:** Publication bias funnel plot.

## Data Availability

The data that supports the findings of this study are available in the supplementary material of this article.
