# Supplementary material for: Comparison of Single Versus Multiple Nonpharmacological Interventions for the Management of Lung Cancer–Related Fatigue: A Systematic Review
Source: Clin Respir J. 2025 Oct 24;19(10):e70132. doi: 10.1111/crj.70132 (PMC12550517; doi:10.1111/crj.70132)
Supplement: Supplementary file 1 — Table S1: PRISMA 2020 checklist. Table S2: Cochrane library search table. Table S3: MEDLINE search table. Table S4: Scopus search table. Table S5: CINHAL search table. Table S6: ProQuest search table. Table S7: Data extraction sheet template. Figure S1: QoL assessment after completion physical activity interventions. Figure S2: QoL assessment after completion traditional Chinese medicine (TCM) interventions. Figure S3: Intervention post assessment. Figure S4: Publication bias funnel plot. [file CRJ-19-e70132-s001.docx]

**Appendix A. Supplementary Data**

Table S1. PRISMA 2020 checklist

| Section and Topic | Item # | Checklist item | Location where item is reported |
| --- | --- | --- | --- |
| TITLE | | |  |
| Title | 1 | Identify the report as a systematic review. | 1 |
| ABSTRACT | | |  |
| Abstract | 2 | See the PRISMA 2020 for Abstracts checklist. | 3 |
| INTRODUCTION | | |  |
| Rationale | 3 | Describe the rationale for the review in the context of existing knowledge. | 6 |
| Objectives | 4 | Provide an explicit statement of the objective(s) or question(s) the review addresses. | 8 |
| METHODS | | |  |
| Eligibility criteria | 5 | Specify the inclusion and exclusion criteria for the review and how studies were grouped for the syntheses. | 9 |
| Information sources | 6 | Specify all databases, registers, websites, organisations, reference lists and other sources searched or consulted to identify studies. Specify the date when each source was last searched or consulted. | 9 |
| Search strategy | 7 | Present the full search strategies for all databases, registers and websites, including any filters and limits used. | 9 |
| Selection process | 8 | Specify the methods used to decide whether a study met the inclusion criteria of the review, including how many reviewers screened each record and each report retrieved, whether they worked independently, and if applicable, details of automation tools used in the process. | 9 |
| Data collection process | 9 | Specify the methods used to collect data from reports, including how many reviewers collected data from each report, whether they worked independently, any processes for obtaining or confirming data from study investigators, and if applicable, details of automation tools used in the process. | 10 |
| Data items | 10a | List and define all outcomes for which data were sought. Specify whether all results that were compatible with each outcome domain in each study were sought (e.g. for all measures, time points, analyses), and if not, the methods used to decide which results to collect. | 10 |
|  | 10b | List and define all other variables for which data were sought (e.g. participant and intervention characteristics, funding sources). Describe any assumptions made about any missing or unclear information. | 10 |
| Study risk of bias assessment | 11 | Specify the methods used to assess risk of bias in the included studies, including details of the tool(s) used, how many reviewers assessed each study and whether they worked independently, and if applicable, details of automation tools used in the process. | 10 |
| Effect measures | 12 | Specify for each outcome the effect measure(s) (e.g. risk ratio, mean difference) used in the synthesis or presentation of results. | 11 |
| Synthesis methods | 13a | Describe the processes used to decide which studies were eligible for each synthesis (e.g. tabulating the study intervention characteristics and comparing against the planned groups for each synthesis (item #5)). | 9 |
|  | 13b | Describe any methods required to prepare the data for presentation or synthesis, such as handling of missing summary statistics, or data conversions. | 10 |
|  | 13c | Describe any methods used to tabulate or visually display results of individual studies and syntheses. | 10 |
|  | 13d | Describe any methods used to synthesize results and provide a rationale for the choice(s). If meta-analysis was performed, describe the model(s), method(s) to identify the presence and extent of statistical heterogeneity, and software package(s) used. | 11 |
|  | 13e | Describe any methods used to explore possible causes of heterogeneity among study results (e.g. subgroup analysis, meta-regression). | 11 |
|  | 13f | Describe any sensitivity analyses conducted to assess robustness of the synthesized results. | 11 |
| Reporting bias assessment | 14 | Describe any methods used to assess risk of bias due to missing results in a synthesis (arising from reporting biases). | 10 |
| Certainty assessment | 15 | Describe any methods used to assess certainty (or confidence) in the body of evidence for an outcome. | 11 |
| RESULTS | | |  |
| Study selection | 16a | Describe the results of the search and selection process, from the number of records identified in the search to the number of studies included in the review, ideally using a flow diagram. | 11 |
|  | 16b | Cite studies that might appear to meet the inclusion criteria, but which were excluded, and explain why they were excluded. | 12 |
| Study characteristics | 17 | Cite each included study and present its characteristics. | 12-17 |
| Risk of bias in studies | 18 | Present assessments of risk of bias for each included study. | 18 |
| Results of individual studies | 19 | For all outcomes, present, for each study: (a) summary statistics for each group (where appropriate) and (b) an effect estimate and its precision (e.g. confidence/credible interval), ideally using structured tables or plots. | 19-24 |
| Results of syntheses | 20a | For each synthesis, briefly summarise the characteristics and risk of bias among contributing studies. | 12-17 |
|  | 20b | Present results of all statistical syntheses conducted. If meta-analysis was done, present for each the summary estimate and its precision (e.g. confidence/credible interval) and measures of statistical heterogeneity. If comparing groups, describe the direction of the effect. | 19-24 |
|  | 20c | Present results of all investigations of possible causes of heterogeneity among study results. | 19-24 |
|  | 20d | Present results of all sensitivity analyses conducted to assess the robustness of the synthesized results. | 19-24 |
| Reporting biases | 21 | Present assessments of risk of bias due to missing results (arising from reporting biases) for each synthesis assessed. | 18-19 |
| Certainty of evidence | 22 | Present assessments of certainty (or confidence) in the body of evidence for each outcome assessed. | 19-24 |
| DISCUSSION | | |  |
| Discussion | 23a | Provide a general interpretation of the results in the context of other evidence. | 24-29 |
|  | 23b | Discuss any limitations of the evidence included in the review. | 24-29 |
|  | 23c | Discuss any limitations of the review processes used. | 24-29 |
|  | 23d | Discuss implications of the results for practice, policy, and future research. | 24-29 |
| OTHER INFORMATION | | |  |
| Registration and protocol | 24a | Provide registration information for the review, including register name and registration number, or state that the review was not registered. | 9 |
|  | 24b | Indicate where the review protocol can be accessed, or state that a protocol was not prepared. | - |
|  | 24c | Describe and explain any amendments to information provided at registration or in the protocol. | 2 |
| Support | 25 | Describe sources of financial or non-financial support for the review, and the role of the funders or sponsors in the review. | 2 |
| Competing interests | 26 | Declare any competing interests of review authors. | 2 |
| Availability of data, code and other materials | 27 | Report which of the following are publicly available and where they can be found: template data collection forms; data extracted from included studies; data used for all analyses; analytic code; any other materials used in the review. | 30 |

*From:*  Page MJ, McKenzie JE, Bossuyt PM, Boutron I, Hoffmann TC, Mulrow CD, et al. The PRISMA 2020 statement: an updated guideline for reporting systematic reviews. BMJ 2021;372:n71. doi: 10.1136/bmj.n71 For more information, visit: <http://www.prisma-statement.org/>

Table S2. Cochrane library search table

| Database  Cochrane library | Group | Search syntax | Number of articles |
| --- | --- | --- | --- |
|  | #1 | ("Lung Neoplasms" OR "Adenocarcinoma of Lung" OR "Adenocarcinoma, Bronchiolo-Alveolar" OR "Bronchial Neoplasms" OR "Mesothelioma, Malignant" OR "Multiple Pulmonary Nodules" OR "Carcinoma, Non-Small-Cell Lung" OR "Small Cell Lung Carcinoma") OR ("Lung Neoplasms" OR "Pulmonary Neoplasm" OR "Pulmonary Neoplasms" OR "Lung Cancer" OR "Lung Cancers" OR "Pulmonary Cancer" OR "Pulmonary Cancers" OR "Cancer of Lung" OR "cancer of the lung" OR "lung tum?or" OR "lung carcinoma"):ti,ab,kw | 25762 |
|  | #2 | ("Fatigue*" OR "Ast?enia" OR Frail* OR Weak* OR Tired* OR wear* OR exhaust* OR apath* OR "letharg*" OR "Cachexia" OR lassitude OR muscle fatigue OR "chronic fatigue syndrome" OR Mobil* limit* OR ambulat* difficulti* OR perceived fatigue):ti,ab,kw | 91292 |
|  | #3 | (("Exercise" or exertion or fitness or "training" or "activity") near/3 ("aerobic" or "physical" or training or isometric or acute or rehabilitat*or muscle resistant move* technique* or breath*)) or (("Therap*" or "medicine*" or technique*) near/3 (alternative or complement* or cup* or soft tissue or mind-body or touch or "cognitive behavioural" or relaxation or herbal or chinese)) or (Stretch* near/3 (dynamic or passive or relaxed)) or (sport* or walk or "yoga" or "Tai chi" or Motor activiti* or Tai ji or QIGONG or Reiki or Massage* or Meditat* or therapeutic touch or "non-pharmacological" or psychotherap*or "Diet*" or "nutrition*" or food or "Acupuncture" or acupressure or Chinese herbal or Herbal drug or Chinese drug):ti,ab,kw | 327429 |
|  | #4 | #1 and #2 and #3 | 444 |
|  | #5 | ("meta-analysis" OR "meta-analyses" OR "systematic review" OR "systematic reviews" OR "a randomised controlled trial"):ti,ab,kw | 129133 |
|  | #6 | #4 and #5 | 73 |

Date filtered from January 2003- January 2023

Table S3. Medline search table

| Database  Medline | Group | Search syntax | Number of articles |
| --- | --- | --- | --- |
|  | 1 | ((Lung* or pleural or Pulmonary or respiratory) adj3 (Neoplasm* or Cancer* or Carcinoma* or Nodule* or Mesothelioma or Adenocarcinoma* or squamous cell* carcinoma* or large cell* carcinoma* or Oat Cell* or Tumo?r*)).ti,ab. | 291,363 |
|  | 2 | exp lung neoplasms/ or exp "adenocarcinoma of lung"/ or exp carcinoma, bronchogenic/ or exp pleural neoplasms/ | 282,351 |
|  | 3 | (((Lung* or pleural or Pulmonary or respiratory) adj3 (Neoplasm* or Cancer* or Carcinoma* or Nodule* or Mesothelioma or Adenocarcinoma* or squamous cell* carcinoma* or large cell* carcinoma* or Oat Cell* or Tumo?r*)).ti,ab.) OR (exp lung neoplasms/ or exp "adenocarcinoma of lung"/ or exp carcinoma, bronchogenic/ or exp pleural neoplasms/) | 387,143 |
|  | 4 | (Fatigue* or Asthenia or Frail* or Weak* or Tired* or wear* or exhaust* or apath* or letharg* or Cachexia or lassitude or cancer related fatigue).ti,ab. | 802,456 |
|  | 5 | Asthenia/ or Fatigue/ | 36,350 |
|  | 6 | (Fatigue* or Asthenia or Frail* or Weak* or Tired* or wear* or exhaust* or apath* or letharg* or Cachexia or lassitude or cancer related fatigue).ti,ab. OR (Asthenia/ or Fatigue/) | 810,561 |
|  | 7 | (((Exercise or exertion or fitness or training or activity) adj3 (aerobic or physical or training or isometric or acute or rehabilitat*or muscle resistant move* technique* or breath*)) or ((Therap* or medicine* or technique*) adj3 (alternative or complement* or cup* or soft tissue or mind-body or touch or cognitive behavioural or relaxation or herbal or chinese)) or (Stretch* adj3 (dynamic or passive or relaxed)) or (sport* or walk or yoga or Tai chi or Motor activiti* or Tai ji or QIGONG or Reiki or Massage* or Meditat* or therapeutic touch or non-pharmacological or psychotherap*or Diet* or nutrition* or food or Acupuncture or acupressure or Chinese herbal or Herbal drug or Chinese drug)).ti,ab. | 1,770,907 |
|  | 8 | therapeutics/ or combined modality therapy/ or electroacupuncture/ or acupuncture therapy/ or medicine, chinese traditional/ or mind-body therapies/ or meditation/ or relaxation therapy/ or tai ji/ or therapeutic touch/ or yoga/ or music therapy/ | 254,857 |
|  | 9 | (((Exercise or exertion or fitness or training or activity) adj3 (aerobic or physical or training or isometric or acute or rehabilitat*or muscle resistant move* technique* or breath*)) or ((Therap* or medicine* or technique*) adj3 (alternative or complement* or cup* or soft tissue or mind-body or touch or cognitive behavioural or relaxation or herbal or chinese)) or (Stretch* adj3 (dynamic or passive or relaxed)) or (sport* or walk or yoga or Tai chi or Motor activiti* or Tai ji or QIGONG or Reiki or Massage* or Meditat* or therapeutic touch or non-pharmacological or psychotherap*or Diet* or nutrition* or food or Acupuncture or acupressure or Chinese herbal or Herbal drug or Chinese drug)).ti,ab. OR (therapeutics/ or combined modality therapy/ or electroacupuncture/ or acupuncture therapy/ or medicine, chinese traditional/ or mind-body therapies/ or meditation/ or relaxation therapy/ or tai ji/ or therapeutic touch/ or yoga/ or music therapy/) | 1,974,040 |
|  | 10 | 3 and 6 and 9 | 984 |
|  | 11 | (Randomi?ed controlled trials or RCT or controlled trials).ti,ab. | 166377 |
|  | 12 | 10 and 11 | 44 |
|  | 13 | Limit 12 to yr=”2003-2023” | 43 |

Date filtered from January 2003- January 2023

Table S4. Scopus search table

| Database  Scopus | Group | Syntax search | Number of articles |
| --- | --- | --- | --- |
|  | 1 | KEY('lung AND tumor'/ OR 'lung AND adenocarcinoma'/ OR 'lung AND alveolus AND cell AND carcinoma'/ OR 'multiple AND pulmonary AND nodules'/ AND tumo?r'/ OR 'lung AND blastoma'/ OR 'pulmonary AND non' AND small AND cell AND lung AND cancer'/ OR 'small AND cell AND lung AND cancer'/) | 29 |
|  | 2 | TITLE-ABS(("Lung Neoplasms" OR "Adenocarcinoma of Lung" OR "Adenocarcinoma, Bronchiolo-Alveolar" OR "Bronchial Neoplasms" OR "lung Mesothelioma" OR "Multiple Pulmonary Nodules" OR "Carcinoma, Non-Small-Cell Lung" OR "Small Cell Lung Carcinoma" OR "Pulmonary Neoplasm" OR "Pulmonary Neoplasms" OR "Lung Cancer" OR "Pulmonary Cancer" OR "Cancer of Lung" OR "cancer of the lung" OR "lung tumo*r" OR "Oat lung cell")) | 254,616 |
|  | 3 | 1 or 2 | 74 |
|  | 4 | KEY('fatigue'/ OR 'asthenia'/ OR 'cancer related fatigue'/ OR 'frailty'/ OR 'cachexia'/ OR 'weakness'/) | 9,894 |
|  | 5 | TITLE-ABS(("Fatigue*" OR "Ast?enia" OR Frail* OR Weak* OR Tired* OR wear* OR exhaust* OR apath* OR "letharg*" OR "Cachexia" OR lassitude OR "chronic fatigue syndrome" OR Mobil* limit* OR ambulat* difficulti* OR perceived fatigue OR “cancer related fatigue”)) | 1,666 |
|  | 6 | 4 or 5 | 11,516 |
|  | 7 | 3 and 6 | 501 |
|  | 8 | KEY('exercise'/ OR 'aerobic exercise'/ OR 'plyometrics'/ OR 'resistance training'/ OR 'stretching'/ OR acute/ OR rehabilitat*/ OR muscle resistant move/ OR physical activiti*/ OR sport*/ OR physical/ OR training*/ OR isometric training*/ OR rehabilitat*/ OR walk/ OR yoga/ OR Tai chi/) | 1 |
|  | 9 | TITLE-ABS(("Exercise" or exertion or fitness or "training" or "activity") W/3 ("aerobic" or "physical" or rehabilitat* OR isometric or acute)) | 325,113 |
|  | 10 | 8 or 9 | 325,114 |
|  | 11 | KEY(Alternative therapi*/ OR Diet*/ OR nutrition*/ OR ‘Acupuncture’/ OR ‘acupressure’/ OR Herbal Medic*/ OR non-pharmacological/ OR cognitive behavioural therap*/ OR Massage/) | 436 |
|  | 12 | TITLE-ABS("Therap*" or "medicine*" or technique*) W/3(alternative or complement* OR mind-body or "cognitive behavioural" or relaxation or herbal) | 202,520 |
|  | 13 | 11 or 12 | 202,735 |
|  | 14 | 7 and 13 | 2 |
|  | 15 | TITLE-ABS(Diet* OR nutrition* OR food OR Diet therapi* OR diet modifi* OR restrict* diet* OR Acupuncture OR acupressure OR Herbal Medic* OR Chinese medic* OR Chinese herbal OR Herbal drug OR Chinese drug) | 49 |
|  | 16 | (TITLE-ABS (( "Lung Neoplasms" OR "Adenocarcinoma of Lung" OR "Adenocarcinoma, Bronchiolo-Alveolar" OR "Bronchial Neoplasms" OR "lung Mesothelioma" OR "Multiple Pulmonary Nodules" OR "Carcinoma, Non-Small-Cell Lung" OR "Small Cell Lung Carcinoma" OR "Pulmonary Neoplasm" OR "Pulmonary Neoplasms" OR "Lung Cancer" OR "Pulmonary Cancer" OR "Cancer of Lung" OR "cancer of the lung" OR "lung tumo*r" OR "Oat lung cell" ) ) OR KEY ( 'lung AND tumor'/ OR 'lung AND adenocarcinoma'/ OR 'lung AND alveolus AND cell AND carcinoma'/ OR 'multiple AND pulmonary AND nodules'/ AND tumo?r'/ OR 'lung AND blastoma'/ OR 'pulmonary AND non' AND small AND cell AND lung AND cancer'/ OR 'small AND cell AND lung AND cancer'/ ) AND TITLE-ABS ( ( "Fatigue*" OR "Ast?enia" OR frail* OR weak* OR tired* OR wear* OR exhaust* OR apath* OR "letharg*" OR "Cachexia" OR lassitude OR "chronic fatigue syndrome" OR mobil* AND limit* OR ambulat* AND difficulti* OR perceived AND fatigue OR "cancer related fatigue" ) ) OR KEY ( 'fatigue'/ OR 'asthenia'/ OR 'cancer AND related AND fatigue'/ OR 'frailty'/ OR 'cachexia'/ OR 'weakness'/ )) and ((KEY('exercise'/ OR 'aerobic exercise'/ OR 'plyometrics'/ OR 'resistance training'/ OR 'stretching'/ OR acute/ OR rehabilitat*/ OR muscle resistant move/ OR physical activiti*/ OR sport*/ OR physical/ OR training*/ OR isometric training*/ OR rehabilitat*/ OR walk/ OR yoga/ OR Tai chi/)) OR (TITLE-ABS(("Exercise" or exertion or fitness or "training" or "activity") W/3 ("aerobic" or "physical" or rehabilitat* OR isometric or acute)))) or ((KEY(Alternative therapi*/ OR Diet*/ OR nutrition*/ OR 'Acupuncture'/ OR 'acupressure'/ OR Herbal Medic*/ OR non-pharmacological/ OR cognitive behavioural therap*/ OR Massage/)) OR (TITLE-ABS("Therap*" or "medicine*" or technique*) W/3(alternative or complement* OR mind-body or "cognitive behavioural" or relaxation or herbal))) or (TITLE-ABS(Diet* OR nutrition* OR food OR Diet therapi* OR diet modifi* OR restrict* diet* OR Acupuncture OR acupressure OR Herbal Medic* OR Chinese medic* OR Chinese herbal OR Herbal drug OR Chinese drug)) | 24 |

Date filtered from January 2003- January 2023

Table S5. CINHAL search table

| Database  CINHAL | Group | Search syntax | Number of articles |
| --- | --- | --- | --- |
|  | 1 | TI (((Lung* or pleural or Pulmonary or respiratory) N3 (Neoplasm* or Cancer* or Carcinoma* or Nodule* or Mesothelioma or Adenocarcinoma* or squamous cell* carcinoma* or large cell* carcinoma* or Oat Cell* or Tumo?r*))) OR AB ( ((Lung* or pleural or Pulmonary or respiratory) N3 (Neoplasm* or Cancer* or Carcinoma* or Nodule* or Mesothelioma or Adenocarcinoma* or squamous cell* carcinoma* or large cell* carcinoma* or Oat Cell* or Tumo?r*))) | 61,987 |
|  | 2 | TI ((Fatigue* or Asthenia or Frail* or Weak* or Tired* or exhaust* or apath* or letharg* or Cachexia or lassitude)) OR AB ((Fatigue* or Asthenia or Frail* or Weak* or Tired* or exhaust* or apath* or letharg* or Cachexia or lassitude)) | 137,014 |
|  | 3 | 1 and 2 | 2,070 |
|  | 4 | TI ((((Exercise or exertion or fitness or training or activity) N3 (aerobic or physical or training or isometric or acute or rehabilitat*or muscle resistant move* technique* or breath*)) or ((Therap* or medicine* or technique*) N3 (alternative or complement* or cup* or soft tissue or mind-body or touch or cognitive behavioural or relaxation or herbal or chinese)) or (Stretch* N3 (dynamic or passive or relaxed)) or (sport* or walk or yoga or Tai chi or Motor activiti* or Tai ji or QIGONG or Reiki or Massage* or Meditat* or therapeutic touch or non-pharmacological or psychotherap*or Diet* or nutrition* or food or Acupuncture or acupressure or Chinese herbal or Herbal drug or Chinese drug)) OR AB ((((Exercise or exertion or fitness or training or activity) N3 (aerobic or physical or training or isometric or acute or rehabilitat*or muscle resistant move* technique* or breath*)) or ((Therap* or medicine* or technique*) N3 (alternative or complement* or cup* or soft tissue or mind-body or touch or cognitive behavioural or relaxation or herbal or chinese)) or (Stretch* N3 (dynamic or passive or relaxed)) or (sport* or walk or yoga or Tai chi or Motor activiti* or Tai ji or QIGONG or Reiki or Massage* or Meditat* or therapeutic touch or non-pharmacological or psychotherap*or Diet* or nutrition* or food or Acupuncture or acupressure or Chinese herbal or Herbal drug or Chinese drug)) | 456,607 |
|  | 5 | 3 and 4 | 264 |
|  | 6 | TI (Randomi?ed controlled Trials OR RCT OR Controlled trials) OR AB (Randomised controlled Trials OR RCT OR Controlled trials) | 175,953 |
|  | 7 | 5 and 6 | 49 |

Date filtered from January 2003- January 2023

Table S6. ProQuest search table

| Database  ProQuest | Group | Search syntax | Number of articles |
| --- | --- | --- | --- |
|  | 1 | abstract(("Lung Neoplasms" OR "Adenocarcinoma of Lung" OR "Adenocarcinoma, Bronchiolo-Alveolar" OR "Bronchial Neoplasms" OR "Mesothelioma, Malignant" OR "Multiple Pulmonary Nodules" OR "Carcinoma, Non-Small-Cell Lung" OR "Small Cell Lung Carcinoma") OR ("Lung Neoplasms" OR "Pulmonary Neoplasm" OR "Pulmonary Neoplasms" OR "Lung Cancer" OR "Lung Cancers" OR "Pulmonary Cancer" OR "Pulmonary Cancers" OR "Cancer of Lung" OR "cancer of the lung" OR "lung tum?or" OR "lung carcinoma")) OR title(("Lung Neoplasms" OR "Adenocarcinoma of Lung" OR "Adenocarcinoma, Bronchiolo-Alveolar" OR "Bronchial Neoplasms" OR "Mesothelioma, Malignant" OR "Multiple Pulmonary Nodules" OR "Carcinoma, Non-Small-Cell Lung" OR "Small Cell Lung Carcinoma") OR ("Lung Neoplasms" OR "Pulmonary Neoplasm" OR "Pulmonary Neoplasms" OR "Lung Cancer" OR "Lung Cancers" OR "Pulmonary Cancer" OR "Pulmonary Cancers" OR "Cancer of Lung" OR "cancer of the lung" OR "lung tum?or" OR "lung carcinoma")) | 137,279 |
|  | 2 | abstract(("Fatigue*" OR "Ast?enia" OR Frail* OR Weak* OR Tired* OR wear* OR exhaust* OR apath* OR "letharg*" OR "Cachexia" OR lassitude OR muscle fatigue OR "chronic fatigue syndrome" OR Mobil* limit* OR ambulat* difficulti* OR perceived fatigue)) OR title (("Fatigue*" OR "Ast?enia" OR Frail* OR Weak* OR Tired* OR wear* OR exhaust* OR apath* OR "letharg*" OR "Cachexia" OR lassitude OR muscle fatigue OR "chronic fatigue syndrome" OR Mobil* limit* OR ambulat* difficulti* OR perceived fatigue)) | 4,115,566 |
|  | 3 | 1 and 2 | 3,206 |
|  | 4 | abstract(Exercise OR physical activiti* OR physical exertion OR physical fitness OR sport* OR aerobic exercise* OR physical exercise* OR exercise training* OR isometric training* OR acute exercise* OR exercise rehabilitat* OR walk OR yoga OR Tai chi OR Motor activiti* OR Tai ji OR muscle stretch* exercise OR dynamic stretch* OR Passive stretch* OR relaxed stretch* OR resistant exercise* OR exercise move* technique* OR breath*exercise OR QIGONG OR Diet* OR nutrition* OR food OR Diet therapi* OR diet modifi* OR restrict* diet* OR Acupuncture OR acupressure OR Herbal Medic* OR Chinese medic* OR Chinese herbal OR Herbal drug OR Chinese drug OR Alternative therapi* OR complement* therapi* OR alternat* medic* OR cup* therapi* OR Relaxation technique* OR Reiki OR Massage OR soft tissue therapi* OR Meditation OR Mind-body therapi* OR touch therapi* OR therapeutic touch OR non-pharmacological OR cognitive behavioural therap* OR cognitive psychotherap*) OR title(Exercise OR physical activiti* OR physical exertion OR physical fitness OR sport* OR aerobic exercise* OR physical exercise* OR exercise training* OR isometric training* OR acute exercise* OR exercise rehabilitat* OR walk OR yoga OR Tai chi OR Motor activiti* OR Tai ji OR muscle stretch* exercise OR dynamic stretch* OR Passive stretch* OR relaxed stretch* OR resistant exercise* OR exercise move* technique* OR breath*exercise OR QIGONG OR Diet* OR nutrition* OR food OR Diet therapi* OR diet modifi* OR restrict* diet* OR Acupuncture OR acupressure OR Herbal Medic* OR Chinese medic* OR Chinese herbal OR Herbal drug OR Chinese drug OR Alternative therapi* OR complement* therapi* OR alternat* medic* OR cup* therapi* OR Relaxation technique* OR Reiki OR Massage OR soft tissue therapi* OR Meditation OR Mind-body therapi* OR touch therapi* OR therapeutic touch OR non-pharmacological OR cognitive behavioural therap* OR cognitive psychotherap*) | 12,857,872 |
|  | 5 | 3 and 4 | 387 |
|  | 6 | ("meta-analysis" OR "meta-analyses" OR "systematic review" OR "systematic reviews" OR "a randomi?ed controlled trial") | 314,762 |
|  | 7 | 5 and 6 | 49 |

Date filtered from January 2003- January 2023

Table S7. Data extraction sheet template

| Name of the study | Authors | Year | Country | Number of participants | Age | Mean age (control group) | Mean age (Intervention group) | Gender | Type of lung cancer | Type of treatment | Fatigue assessment tool | QoL assessment tool | Type of intervention | Single modal or multi-modal | Duration |
| --- | --- | --- | --- | --- | --- | --- | --- | --- | --- | --- | --- | --- | --- | --- | --- |
|  |  |  |  |  |  |  |  |  |  |  |  |  |  |  |  |
|  |  |  |  |  |  |  |  |  |  |  |  |  |  |  |  |
|  |  |  |  |  |  |  |  |  |  |  |  |  |  |  |  |
|  |  |  |  |  |  |  |  |  |  |  |  |  |  |  |  |
|  |  |  |  |  |  |  |  |  |  |  |  |  |  |  |  |
|  |  |  |  |  |  |  |  |  |  |  |  |  |  |  |  |
|  |  |  |  |  |  |  |  |  |  |  |  |  |  |  |  |
|  |  |  |  |  |  |  |  |  |  |  |  |  |  |  |  |
|  |  |  |  |  |  |  |  |  |  |  |  |  |  |  |  |
|  |  |  |  |  |  |  |  |  |  |  |  |  |  |  |  |
|  |  |  |  |  |  |  |  |  |  |  |  |  |  |  |  |
|  |  |  |  |  |  |  |  |  |  |  |  |  |  |  |  |
|  |  |  |  |  |  |  |  |  |  |  |  |  |  |  |  |

Figure S1. QoL assessment after completion physical activity interventions

Figure S2. QoL assessment after completion traditional Chinese medicine (TCM) interventions

Figure S3. Intervention post assessment

Figure S4. Publication bias funnel plot
